# Association of *ABCB1* and *CYP2C19* polymorphisms with major adverse cardiovascular complications in patients taking clopidogrel

**DOI:** 10.3389/fcvm.2026.1844931

**Published:** 2026-06-24

**Authors:** Lubna Q. Khasawneh, Mais N. Alqasrawi, Zeina N. Al-Mahayri, Lilas Dabaghie, Sahar M. Altoum, Gohar Jamil, Salahdein Aburuz, Dana Hamza, Lizy George, Faiz Al-Bakshy, Kaes Al-Anee, Khuzama AlAhamad, Fatma Al-Maskari, Husam Ouda, Juma AlKaabi, George P. Patrinos, Bassam R. Ali

**Affiliations:** 1 Department of Genetics and Genomics, College of Medicine and Health Sciences, United Arab Emirates University, Al-Ain, United Arab Emirates; 2 Department of Biomedical Sciences, College of Health Sciences, Abu Dhabi University, Al-Ain, United Arab Emirates; 3 Pharmacy Department, Sheikh Tahnoon Bin Mohammed Medical City, Al-Ain, United Arab Emirates; 4 Department of Cardiovascular Medicine, Sheikh Tahnoon Bin Mohammed Medical City, United Arab Emirates University, Al-Ain, United Arab Emirates; 5 Department Pharmacology and Therapeutics, College of Medicine and Health Sciences, United Arab Emirates University, Al-Ain, United Arab Emirates; 6 Department of Medicine, National Health Services (NHS), North Bristol Trust (NBT), Southmead Hospital, London, United Kingdom; 7 Department of Cardiology, Mediclinic Al-Ain Hospital, Al-Ain, United Arab Emirates; 8 Department of Clinical Pharmacy, Mediclinic Al-Ain Hospital, Al-Ain, United Arab Emirates; 9 Public Health Institute, College of Medicine and Health Sciences, United Arab Emirates University, Al-Ain, United Arab Emirates; 10 The Heart Medical Center, Al-Ain, United Arab Emirates; 11 Department of Medicine, College of Medicine and Health Sciences, United Arab Emirates University, Al-Ain, United Arab Emirates; 12 School of Health Sciences, Department of Pharmacy, Laboratory of Pharmacogenomics and Individualized Therapy, University of Patras, Patras, Greece; 13 ASPIRE Precision Medicine Research Institute Abu Dhabi, United Arab Emirates University, Al Ain, United Arab Emirates; 14 Zayed Centre for Health Sciences, United Arab Emirates University, Al-Ain, United Arab Emirates

**Keywords:** *ABCB1*, clopidogrel, *CYP2C19*, MACE, United Arab Emirates

## Abstract

**Background:**

The genetic profile may contribute to interindividual differences in clopidogrel response among patients with acute coronary syndrome (ACS). Although *CYP2C19* variants have documented established recommendations that they have contributed to reducing the efficacy of medication, the clinical impact of *ABCB1* polymorphisms on clopidogrel bioavailability and subsequent clinical outcome remains controversial. This study evaluates the role of *ABCB1* and *CYP2C19* variants with major adverse cardiovascular events (MACE) in patients with ACS in a sample obtained from the United Arab Emirates (UAE).

**Methods:**

This retrospective cohort study included 174 patients with ACS treated with clopidogrel. Genotyping for *ABCB1* and *CYP2C19* variants was performed using real-time PCR® TaqMan assays. Associations with 1-year MACE outcome were assessed using genotype, carrier status, and dominant and recessive models. A multivariable logistic regression analysis was also conducted.

**Results:**

The minor allele frequencies for the *ABCB1* gene variants, rs1045642, rs2032582, and rs1128503, were 48.85%, 50%, and 51.44%, respectively. The presence of *ABCB1* alleles had no significant correlation outcomes with MACE, regardless of the genetic models used (*p*-value > 0.05). CYP2C19*2 was associated with a weak trend of increased risk of MACE in the dominant model (RR = 1.41; 95% CI: 0.95–2.10; *p*-value = 0.08), whereas the rare CYP2C19*3 (1.15%) was not significantly related to any of the outcomes. In multivariable logistic regression, only age showed a non-significant trend toward higher risk.

**Conclusion:**

The study did not find any significant link between common *ABCB1* variants and MACE in patients with ACS treated with clopidogrel from the UAE sample. None of these variants were found to be predictors of outcomes after a multivariate analysis was performed. These findings suggest limited current utility for *ABCB1* variants in clopidogrel response.

## 1 Introduction

It is widely recognized that *CYP2C19* polymorphisms play an important role in determining variations in individual response to clopidogrel treatment, with Loss-Of-Function (LOF) allele mutations (e.g., CYP2C19*2 and CYP2C19*3) causing inadequate platelet suppression and an elevated likelihood of adverse cardiovascular outcomes (1–3). Therefore, in the current clinical practice guidelines, such as those provided by the Clinical Pharmacogenetics Implementation Consortium (CPIC), recommendations have been issued regarding the use of antiplatelet agents based on *CYP2C19* genotyping, especially among patients who have undergone a percutaneous coronary intervention (PCI), wherein other P2Y12 inhibitors (i.e., ticagrelor) might be more suitable in LOF allele carriers (4, 5). Thus, CYP2C19 remains the most clinically established pharmacogenetic marker for clopidogrel therapy (6, 7).

Beyond hepatic metabolism, oral drug bioavailability is affected by the drug transporters in the intestine (8). In this context, the ATP-binding cassette subfamily B member 1 (ABCB1) and P-glycoprotein (P-gp) have been shown to play a key role in the transport and disposition of drugs within the body (9). Therefore, as a consequence, the role of the protein in drug bioavailability has received considerable interest, especially for the antiplatelet drug clopidogrel, which is prescribed to prevent cardiovascular complications among patients with atherosclerotic cardiovascular diseases (10, 11). Being an orally administered drug, its bioavailability is determined not only by its metabolism in the liver but also by its absorption in the intestines (12). Thus, genetic variations of *ABCB1* might affect clopidogrel pharmacokinetics and subsequent clinical response (13).

Although a large number of variants have been identified (approximately 62) within the coding sequence of the *ABCB1* gene (14), only three common single-nucleotide polymorphisms (SNPs) have received the greatest attention among researchers in cardiovascular pharmacogenomics, and these are rs1128503 (c.C1236T), rs2032582 (c.G2677T/A), and rs1045642 (c.C3435T) (15, 16). These variants have been noted to be in high linkage disequilibrium (LD) and have been associated with changes in P-gp expression or transport activity (17, 18). Some synonymous variants (such as rs1045642) can also influence mRNA stability, translation efficiency, and even protein folding, thereby affecting the functionality of transporters (19). Frequencies of these polymorphisms differ substantially across various ethnicities, including Middle East populations such as those from the UAE (20–22).

However, despite growing scientific interest, the role of *ABCB1* genotyping in patient care remains controversial (23). This is in contrast to *CYP2C19* genotyping, wherein no recommendations for *ABCB1* genotyping in clinical practice can be found in existing literature because of the inconsistencies and contradictions reported in different studies on the association between *ABCB1* and clopidogrel exposure, reactivity of platelets, and clinical outcomes (24) Accordingly, *ABCB1* remains an evolving area of pharmacogenomic research rather than an established clinical biomarker.

Given the limited and heterogeneous data available in Middle East populations, especially in patients from the UAE, further investigations are warranted. Therefore, the current study was conducted to examine the role of common *ABCB1* variants in the risk of the development of major adverse cardiovascular events (MACE) over a period of one year in patients with acute coronary syndrome (ACS) who were on clopidogrel treatment. The relationships were assessed based on carrier status, distribution of genotypes, and allele frequencies. Furthermore, common variants of *CYP2C19* LOF polymorphisms, which were previously linked to poor clopidogrel response, were analyzed to obtain a more complete genetic profile. Moreover, both dominant and recessive inheritance models were applied to perform the analysis, as well as multivariable logistic regression, to explore the role of genetic factors in MACE development.

## 2 Methods

### 2.1 Study design and participants

In an observational retrospective cohort study, 174 (*n* = 174) patients with acute ACS were recruited from a subsequent cohort of patients with cardiovascular diseases. Participants were recruited from three clinical sites: Tawam Hospital, the Medical Heart Center, and Mediclinic Al-Ain Hospital, Al-Ain, UAE. The enrollment of patients was conducted from 28 October 2021 to 14 March 2023. The inclusion criteria were as follows: (1) All patients were diagnosed as patients with ACS based on the recent ACC/AHA guidelines (25). Patient diagnosis was documented in the hospital's electronic system. (2) All patients underwent a procedure of PCI and received clopidogrel as a loading dose of 300 mg and an aspirin dose of 300 mg. Then, the patients received a maintenance dose of clopidogrel (75 mg) and aspirin. The exclusion criteria were as follows: (1) Patients with severe liver or hepatic impairment, (2) patients taking other anticoagulants, (3) patients with active bleeding or having signs of bleeding, (4) patients with a current active tumor or are on chemotherapy treatment, and (5) patients who are known to have hematologic disorders. All patients signed a consent form in compliance with the declaration of Helsinki. The study was approved by “The Abu-Dhabi Health Research and Technology Ethical Committee” with reference number (DOH/CVDC/2020/1187).

### 2.2 Blood sampling and genotyping

Peripheral blood samples were collected in 3 mL EDTA vacuum tubes (BD, USA). The samples were stored at −20 °C. Genomic DNA was isolated from the whole blood using the QIAamp® DNA Kit and FlexiGene® DNA Kit (Qiagen, Germany), following the manufacturer's recommendations. The quality and quantity of the isolated DNA samples were confirmed by using the Nanodrop One Spectrophotometer (Thermo Fisher Scientific, USA). The three *ABCB1* gene polymorphisms, namely, rs1045642 (c.C3435T), rs2032582 (c.G2677T), and rs1128503 (c.C1236T), were detected by using TaqMan® SNP assays ((C___7586657_20, C_11711720C_30, and C___7586662_10, respectively), and the *CYP2C19* variants, namely, rs4244285 (*2) and rs4986893 (*3) (C__25986767_70 and C__27861809_10, respectively), were also detected. Genotyping was performed using TaqMan SNP assays on the QuantStudio 7 Flex Real-Time PCR System (Applied Biosystems, ThermoFisher Scientific, USA). The results were confirmed by using the TaqMan Genotyper Software, version 1.6.0 (Applied Biosystems, ThermoFisher Scientific, USA).

### 2.3 Data collection and follow-up

The demographics of the recruited patients including their age, gender, height, weight, smoking status, and disease history, including diabetes mellitus type 2 and hypertension, and their concomitant medications, were recorded.

The study subjects were retrospectively followed up by collecting data from their medical records, ensuring that no data were missing. The study subjects were on a daily oral dose of 75 mg of clopidogrel for one year, and all were on this dose as part of their secondary prevention strategy for ACS. The study outcomes include the development of MACE as previously defined (26), which include all-cause death, non-fatal myocardial infarction (MI) that has been diagnosed as ST-segment elevated MI (STEMI) or non-ST-segment elevation MI (NSTEMI), unstable angina with PCI, and stroke. The study outcomes include all deaths, and if cardiovascular, then no further etiology is needed.

The study outcomes and MACE events were scored from day one after the incidence of ACS until one year of retrospective data collection without any duplication of the data from the same patient.

All information was recorded using the Castor-EDC® Software (Netherlands) (https://www.castoredc.com). Castor EDC is an online-based solution for collecting data directly from a clinical site using an electronic Case Report Form (eCRF) at clinical sites, which is then stored in a centralized database. Only authorized personnel have access to the software by entering their log-in information to maintain the confidentiality of the research participant's information. Moreover, the Castor-EDC® platform made it easy to export and analyze the research data, which increased the efficiency of the research analysis.

### 2.4 Statistical analysis

The continuous variables were expressed as mean values and categorical variables were expressed as numerical and percentage values. Relative risk (RR) was calculated, and the two-tailed probability value was considered statistically significant at <0.05. To account for multiple comparisons, *p*-values were adjusted using both the Bonferroni correction and the Benjamini–Hochberg false discovery rate (FDR) method, and adjusted *p*-values were reported alongside the uncorrected values.

A haplotypes analysis was carried out using Haploview 4.1 software, and LD was assessed between the selected SNPs from the *ABCB1* gene by calculating D′ and *r*^2^.

A logistic regression analysis was carried out using IBM SPSS statistics software version 29.0.2 (Armonk, NY, USA). A multivariate regression analysis was used to adjust for potential confounding variables, which may predict the outcome.

## 3 Results

### 3.1 Baseline demographics and clinical characteristics of the participants

A total of 174 patients were successfully enrolled and included in the study and were retrospectively followed up for one year. The collected baseline characteristics include age, sex, body mass index (BMI), smoking status, history of diabetes mellitus or hypertension, and concomitant medication. The characteristics of the cohort are presented in Table 1.

**Table 1:** Baseline demographics and characteristics of the study patients.

Baseline characteristics	ACS cohort (*n* = 174)
Age (mean)	55.9
Gender (male/female)	155/19
BMI	
<25	58 (33.33%)
25–30	70 (40.23%)
>30	46 (26.44%)
Cigarette smoking status^a^	
Never smoked	84 (49.7%)
Former smoker	20 (11.8%)
Current smoker	60 (35.5%)
Type of MI	
STEMI	48 (27.7%)
NSTEMI	73 (42%)
Unstable angina	53 (30.3%)
Other diseases (i.e., risk factors)	
Hypertension	121 (69.54%)
Diabetes	97 (55.75%)
Hypercholesterolemia	102 (58.62%)
Concomitant medications	
ACEi/ARBs	100 (57.5%)
ARBs	24 (13.8%)
Statins	173 (99.4%)
Oral antidiabetics	99 (56.9%)
Diuretics	61 (35.1%)
PPIs (pantoprazole)	136 (78.16%)
MACE outcomes	
Developed MACE	30 (17.2%)
No MACE	144 (82.76%)

a 5 missing data, 5 smoke only shisha.

BMI, body mass index; ACEi, angiotensin-converting enzyme inhibitors; ARBs, angiotensin II receptor blockers; PPIs, proton pump inhibitors.

The average age of the individuals with ACS was 55.9 years, and most of the participants were males (*n* = 155). Notably, approximately 26.44% of patients were classified as obese, whereas 40.23% were overweight. For smoking status, current smokers are twice as many patients compared with former smokers (35.5% and 11.8%, respectively). However, half of the patients with ACS never smoked cigarettes (*n* = 84, 49.7%). With regard to risk factors, hypertension, diabetes, and hypercholesterolemia in patients with MI were present in 69.54%, 55.75%, and 58.62% of patients, respectively. In terms of concomitant medications, more than 99% of the cohort was on statins. However, there was a wide range of use of other drugs such as ACE inhibitors, ARBs, diabetic drugs, diuretics, and PPIs among the patients included in the study.

### 3.2 Linkage disequilibrium among the three *ABCB1* gene variants

**Figure 1:**
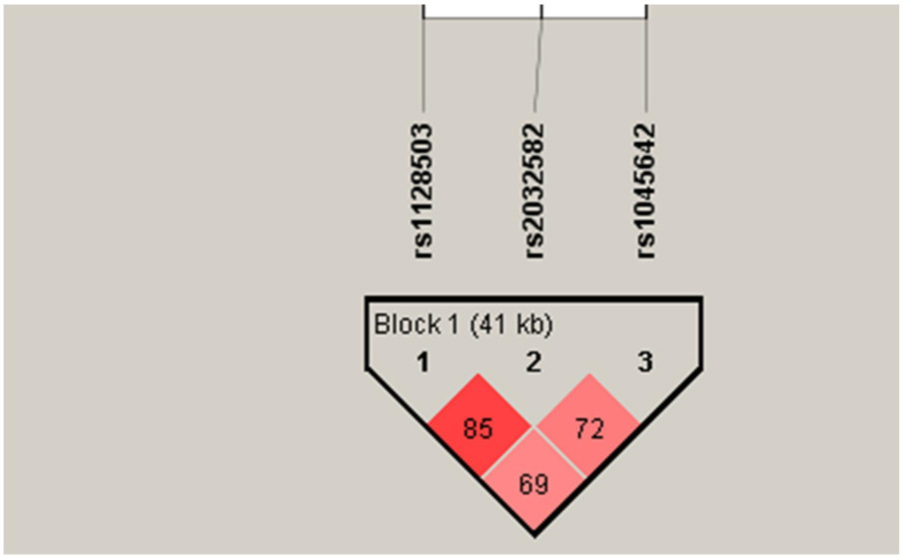
LD among the three *ABCB1* variants (rs1045642, rs2032582, and rs1128503).

An analysis of LD among the three *ABCB1* variants (rs1045642, rs1128503, and rs2032582) revealed that there was a moderate to high degree of non-random association among their alleles in the locus. According to the D′ values detected using the haploview program, the presence of the strongest degree of LD is typical of the rs1128503 and rs2032582 variants (D′ = 85) with the following rs2032582 and rs1045642 variants (D′ = 72) and a moderate degree of LD between rs1128503 and rs1045642 (D′ = 69). Thus, these findings indicate limited historical recombination among the three SNPs and therefore tend to be frequently inherited together as part of common haplotypes. Nevertheless, an analysis of *r*^2^ revealed a different tendency, with the most significant correlation between rs1128503 and rs2032582 variants (*r*^2^ = 69), whereas a low-level correlation characterized rs2032582 and rs1045642 (*r*^2^ = 49) and rs1128503 and rs1045642 (*r*^2^ = 44). This discrepancy between D′ and *r*^2^ is expected, as D′ represents a history of recombination, while *r*^2^ measures the predictive ability of one SNP for another. Overall, from the results, it can be stated that rs1128503 and rs2032582 are the most closely linked variants and may provide overlapping information in the genetic background, while rs1045642 retains partial independent information. The results are shown in Figure 1.

### 3.3 *CYP2C19* and *ABCB1* variant distribution of genotypes and alleles among the cohort

Table 2 presents the distribution of genotypes and alleles for the three *ABCB1* variants as well as the two *CYP2C19* LOF alleles (i.e., CYP2C19*2 and CYP2C19*3) within the current cohort. For the *ABCB1* variants, the rates of frequency of the mutant allele, T allele, from the three variants among the cohort are 48.86% [rs1045642(C3435T)], 50% [rs2032582**(**G2677T)], and 51.44% [rs1128503(C123T)], respectively. The findings revealed that the percentages of individuals carrying one or two minor alleles for each variant were similar among patients with ACS.

**Table 2:** Distribution of the *CYP2C19* and *ABCB1* genotypes and alleles in patients with ACS.

*ABCB1* genotypes			
	*N* (%) (*n* = 174)	*ABCB1* alleles	*N* (%) (*n* = 348)
***ABCB1* gene**			
**rs1045642 (C3435T)**		**rs1045642 (C3435T)**	
CC	50 (28.74)	C allele	178 (51.15)
CT	78 (44.83)	T allele	170 (48.85)
TT	46 (26.44)		
CC vs. CT + TT	50/124		
CC + CT vs. TT	128/46		
**rs2032582 (G2677T)**		**rs2032582** **(G2677T)**	
GG	52 (29.9)	G allele	174 (50)
GT	70 (40.23)	T allele	174 (50)
TT	52 (29.9)		
GG vs. GT + TT	52/122		
GG + GT vs. TT	122/52		
**rs1128503 (C123T)**		**rs1128503** **(C123T)**	
CC	45 (25.86)	C allele	169 (48.56)
CT	79 (45.4)	T allele	179 (51.44)
TT	50 (28.74)		
CC vs. CT + TT	45/129		
CC + CT vs. TT	124/50		
***CYP2C19* gene**			
**CYP2C19 genotypes**		**CYP2C19 alleles**	
rs4244285 (*2)		rs4244285 (*2)	
CC	99 (56.9%)	C allele	261 (75%)
CT	63 (36.2%)	T allele	87 (25%)
TT	12 (6.9%)		
CC vs. CT + TT	99/75		
CC + CT vs. TT	162/12		
rs4986893 (*3)		rs4986893 (*3)	
CC	170 (97.7%)	C allele	344 (98.85%)
CT	4 (2.3%)	T allele	4 (1.15%)
TT	0		
CC vs. CT + TT	170/4		
CC + CT vs. TT	174/0		

However, an analysis of the *CYP2C19* variants showed that the rs4244285*2 LOF allele was relatively common in the cohort, with 43.1% (*n* = 75) of patients carrying at least one variant allele (i.e., 36.2% and 6.9%) and an allele frequency of 25%. In contrast, the rs4986893*3 variant was rare, identified in only 2.3% (*n* = 4) of patients in the heterozygous state (i.e., CT genotype), with no homozygous carriers observed and an allele frequency of 1.15% (*n* = 4, T allele).

The percentages were consistent across genotypes, alleles, and among the dominant and recessive models for each variant.

### 3.4 Comprehensive analysis for the association of the study variants on the risk of MACE among patients with ACS

#### 3.4.1 Risk of MACE based on the study variants’ carrier status

Table 3 demonstrates the association of *ABCB1* and *CYP2C19* gene variants with the occurrence of MACE in patients with ACS. From the results, it is evident that there is no significant variation in the occurrence of thrombotic complications in patients carrying the mutant allele (16.8%, 16.3%, and 16.9%) compared with non-carriers (18.4%, 19.6%, and 18.2%) from any of the three *ABCB1* variants, respectively. However, for *CYP2C19* variants, 22.7% (*n* = 17) of participants who were carriers of the CYP2C19*2 mutant allele developed MACE compared with 13.1% (*n* = 13) non-carriers of the mutant allele.

**Table 3:** Risk of MACE outcome in patients with ACS based on the study variants’ carrier status.

Study variants	Carriers for the mutant allele *N* (%)	Non-carriers (wild type) *N* (%)	RR (95% CI)	*p*-value	Bonferroni-adjusted *p*-value	FDR-adjusted *q*-value
rs1045642	*N* = 125	*N* = 49	0.92 (0.45–1.86)	0.8	1.00	0.85
MACE	21 (16.8)	9 (18.4)				
No MACE	104 (83.2)	40 (81.6)				
rs2032582	*N* = 123	*N* = 51	0.83 (0.42–1.65)	0.6	1.00	0.85
MACE	20 (16.3)	10 (19.6)				
No MACE	103 (83.74)	41 (80.4)				
rs1128503	*N* = 130	*N* = 44	0.93 (0.45–1.94)	0.85	1.00	0.85
MACE	22 (16.9)	8 (18.2)				
No MACE	108 (83.1)	36 (81.8)				
rs4244285 (*2)	*N* = 75	*N* = 99	1.41 (0.95–2.1)	0.08	0.32	0.32
MACE	17 (22.7)	13 (13.1)				
No MACE	58 (77.3)	86 (86.9)				
rs4986893 (*3)	*N* = 4	*N* = 170	N/A	N/A	N/A	N/A
MACE	0	0				
No MACE	4 (100)	170 (100)				

RR, relative risk; FDR, Benjamini–Hochberg False Discovery Rate; the chi-square cutoff value for the *P*-value was less than 0.05, and therefore, it was not considered statistically significant.

The results have also been statistically analyzed to confirm the absence of variations in the risk of cardiovascular complications (*p*-value > 0.05). Raw *p*-values were adjusted using Bonferroni and FDR methods and they remain non-significant.

#### 3.4.2 Risk of MACE based on the study variants’ genotypes

Table 4 presents an analysis of the risk of severe cardiovascular events in patients with ACS based on the genotypes of the *ABCB1* gene and *CYP2C19* gene. The results showed that cardiovascular complications, such as MACE, were more common in ACS patients with heterozygous or homozygous mutant genotypes of any of the three variants of the *ABCB1* gene compared with those with the wild genotype—i.e., 66.6% [i.e., 43.3% (CT) + 23.3% (TT)] compared with 33.3% (CC genotype) for the rs1045642 variant, 62.3% [i.e., 36.6% (GT) + 26.7% (TT)] compared with 36.6% (GG genotype) for the rs2032582 variant, and 73.34% [i.e., 46.67% (GT) + 26.67% (TT)] compared with 26.67% (GG genotype) for the rs1128503 variant, respectively. The percentages were similar when compared with patients with ACS who did not experience cardiovascular complications.

**Table 4:** Risk of MACE outcomes in ACS based on the study variant genotypes.

*ABCB1* variants	MACE (*n* = 30) *N* (%)	No MACE (*n* = 144) *N* (%)	RR (95% CI)	*p*-value	Bonferroni-adjusted *p*-value	FDR-adjusted *q*-value
**rs1045642**						
CC	10 (33.3)	40 (27.8)	Reference			
CT	13 (43.3)	65 (45.12)	0.96 (0.45–2.1)	0.93	1.00	0.93
TT	7 (23.3)	39 (27.08)	0.83 (0.34–2.04)	0.68	1.00	0.87
**rs2032582**						
GG	11 (36.6)	41 (28.47)	Reference			
GT	11 (36.6)	59 (41)	0.86 (0.4–1.84)	0.7		0.87
TT	8 (26.7)	44 (30.56)	0.78 (0.34–1.83)	0.57		0.87
**rs1128503**						
CC	8 (26.67)	36 (25)	Reference			
CT	14 (46.67)	66 (45.83)	0.96 (0.44–2.11)	0.92	1.00	0.93
TT	8 (26.67)	42 (29.17)	0.88 (0.36–2.15)	0.78	1.00	0.88
rs4244285 (*2)						
CC	13 (43.3)	86 (59.7)	Reference			
CT	14 (46.7)	49 (34)	1.62 (0.73–3.6)	0.24	1.00	0.48
TT	3 (10)	9 (6.3)	1.52 (0.37–6.16)	0.56	1.00	0.87
rs4986893 (*3)						
CC	30 (100)	144 (97.2)	Reference	–	–	–
CT	0	4 (2.8)	N/A	N/A	–	–
TT	0	0	N/A	N/A	–	–

For *CYP2C19* genotypes, CYP2C19*2 followed the same pattern as the *ABCB1* variants, as MACE occurred in 56.7% [i.e., 46.7 (CT) + 10% (TT)] of patients with heterozygous or homozygous mutant genotypes compared with those with the wild genotype [i.e., 43.3% (CC genotype)].

Genotype-based analyses showed no statistically significant association between the investigated variants and MACE. After adjustment for multiple comparisons using both Bonferroni and FDR methods, all findings remained non-significant (i.e., *p* > 0.05).

#### 3.4.3 Association of MACE with study variants under dominant and recessive model analyses

According to analyses using the dominant and recessive models presented in Table 5, none of the variants of the *ABCB1* gene examined (rs1045642, rs2032582, and rs1128503) were associated with an increased risk of MACE within one year, as their relative risks were close to 1 and *p*-values were insignificant. Likewise, neither the CYP2C19*2 (rs4244285) variant nor the CYP2C19*3 (rs4986893) variant showed any significant associations with the outcome after a multiple testing correction. Nevertheless, carriers of the CYP2C19*2 allele under the dominant model displayed a non-significant trend toward an increased risk of MACE (RR:1.41, 95% CI: 0.95–2.10; *p* = 0.08). In summary, no significant associations were detected between *ABCB1* and *CYP2C19* variants and adverse cardiac events using either genetic model.

**Table 5:** Association of study variants with MACE under dominant and recessive models.

Variant	Genetic model comparison	MACE *n* (%)	No MACE *n* (%)	RR (95% CI)	*p*-value	Bonferroni-adjusted *p*-value	FDR *q*-value
rs1045642	Dominant (CT + TT vs. CC)	20/30 (66.7%) vs. 10/30 (33.3%)	104/144 (72%) vs. 40/144 (27.8%)	0.92 (0.45–1.86)	0.8	1.00	0.89
	Recessive (TT vs. CC + CT)	7/30 (23.3%) vs. 23/30 (76.7%)	39/144 (27.1%) vs. 105/144 (72.9%)	0.93 (0.45–1.94	0.85	1.00	0.89
rs2032582	Dominant (GT + TT vs. GG)	19/30 (63.3%) vs. 11/30 (36.7%)	103/144 (71.5%) vs. 41/144 (28.5%)	0.83 (0.42–1.65)	0.6	1.00	0.89
	Recessive (TT vs. GG + GT)	8/30 (26.7%) vs. 22/30 (73.3%)	44/144 (30.6%) vs. 100/144 (69.4%)	0.93 (0.45–1.94)	0.85	1.00	0.89
rs1128503	Dominant (CT + TT vs. CC)	22/30 (73.3%) vs. 8/30 (26.7%)	106/144 (75%) vs. 36/144 (25%)	0.93 (0.45–1.94)	0.85	1.00	0.89
	Recessive (TT vs. CC + CT)	8/30 (26.7%) vs. 22/30 (73.3%)	42/144 (29.2%) vs. 102/144 (70.8%)	0.95 (0.46–1.98)	0.89	1.00	0.89
rs4244285 (*2)	Dominant (CT + TT vs. CC	17/30 (56.7%) vs. 13/30 (43.3%)	58/144 (40.3%) vs. 86/144 (59.7%)	1.41 (0.95–2.1)	0.08	0.96	0.48
	Recessive (TT vs. CC + CT)	3/30 (10.0) vs. 27/30 (90.0)	9/144 (6.3) vs. 135/144 (93.7)	1.6 (0.46–5.59)	0.46	1.00	0.89
rs4986893 (*3)	Dominant (CT + TT vs. CC	0/30 (0) vs. 30/30 (100)	4/144 (2.8) vs. 140/144 (97.2)	N/A	0.57	N/A	N/A
	Recessive (TT vs. CC + CT)	No TT genotype	N/A	N/A	N/A	N/A	N/A

**Table 6:** Logistic regression for risk factors associated with MACE events in the current cohort.

Model/variable	Adjusted OR (95% CI)	*p*-value
**Model 1: rs1045642**		
rs1045642 carrier	0.98 (0.4–2.38)	0.96
Age	1.03 (0.99–1.08)	0.11
Male sex	0.56 (0.18–1.74)	0.31
Diabetes	1.35 (0.56–3.25)	0.51
**Model 2: rs2032582**		
rs2032582 carrier	0.82 (0.35–1.93)	0.66
Age	1.03 (0.99–1.08)	0.11
Male sex	0.56 (0.18–1.75)	0.32
Diabetes	1.35 (0.56–3.24)	0.5
**Model 3: rs1128503**		
rs1128503 carrier	0.88 (0.36–2.16)	0.78
Age	1.03 (0.99–1.08)	0.1
Male sex	0.56 (0.18–1.77)	0.33
Diabetes	1.36 (0.56–3.25)	0.5

### 3.5 Multivariable logistic regression analysis for predictors of MACE

None of the investigated *ABCB1* variants were independently linked to the risk of the development of MACE in the multivariable logistic regression analysis presented in Table 6. Even while there was a trend toward increased risk with age, this association did not achieve statistical significance. Similarly, neither sex nor diabetes mellitus were found to be independent predictors of MACE. Overall, the results indicated that neither the included clinical variables nor the studied *ABCB1* polymorphisms had a substantial impact on the cohort's one-year cardiovascular outcomes.

## 4 Discussion

The variation of outcomes in clopidogrel is a well-recognized phenomenon, and the various factors contributing to interindividual variations have been studied worldwide (27). Among the various genetic factors studied, the role of *CYP2C19* LOF alleles is well established by CPIC recommendations since 2022 and it is currently incorporated in the clinical guidelines (28). However, the *ABCB1* pharmacogene has also been studied since 2001 to understand its role in the transportation of clopidogrel (15) and its impact on modulating the risk of the development of cardiovascular complications. Thus, the present study was conducted to assess the relationship of the common *ABCB1* variants along with *CYP2C19* LOF alleles with the occurrence of subsequent MACE in patients with ACS in a sample from the UAE. These findings add to the ongoing debate regarding the clinical significance of *ABCB1* pharmacogenomics in clopidogrel-treated patients.

The rates of prevalence of the three variants of the *ABCB1* gene, namely, rs1045642 (C3435T), rs2032582 (G2677T), and rs1128503 (C123T), in the present study were significantly high, i.e., 48.85%, 50%, and 51.44%, respectively. These frequencies were higher than those reported in previous studies conducted in Saudi Arabia, where the prevalence rates of the *ABCB1* variants were 37%, 35.3%, and 40%, respectively (29). However, the prevalence of the variants of the *ABCB1* gene in the current study was lower than those documented from previous investigations conducted in Jordan (58%, 58%, and 56.5%, respectively) (30).

These three *ABCB1* variants have been implicated in various cardiovascular conditions such as hypertension (31, 32), coronary artery diseases (33), and responses to various cardiovascular medications such as losartan (34), statins (35, 36), digoxin (14), and rivaroxaban (37, 38). However, the relationship between these drugs and cardiovascular outcomes has been found to be inconsistent (15, 39). Despite the conduct of several genome-wide association studies and different clinical studies among diverse populations worldwide, the results have often found to be contradictory.

In relation to clopidogrel-treated ACS patients, interest in the effect of these *ABCB1* variants on cardiovascular outcome was noted. It was established that there was a significant correlation between the *ABCB1* rs1045642 (c.C3435T) TT genotype and clopidogrel treatment response due to an increased risk of occurrence of MACE in comparison with the CC wild-type genotype in patients with ACS (40, 41). However, not all studies were able to show this evidence. This was also evident in the meta-analysis carried out by Zahi and coworkers (42) and in the current study wherein the *p*-value was more than 0.05. On the other hand, this was in agreement with previous studies in relation to *ABCB1* rs102032582 (c.G2667T). This variant was not found to be significantly associated with an increased risk of recurrent stent thrombosis, a major feature in MACE occurrence in patients with ACS. This was also evident in a case–control study conducted by Dong-Yi and coworkers, wherein the *p*-value was 0.793 in relation to patients with ACS and those undergoing PCI (43). In addition, the study conducted by Redig and coworkers that focused on the evaluation of platelet function and the incidence of MACE in patients suffering from angina pectoris further supported the lack of association with clopidogrel responsiveness (44).

Furthermore, 180 patients with ACS were studied to investigate the effect of the rs1128503 (c.C123T) variant on clopidogrel resistance. The variant was found to result in increased platelet inhibition in patients with CT and TT genotypes compared with those with the wild-type genotype (i.e., CC) (45). Conversely, no correlation was observed between the variant genotype and clopidogrel efficacy. This was in agreement with another study on 106 patients with ACS using clopidogrel, wherein the *p*-value was 0.253 (46). Thus, our findings support the latter evidence and suggest that the impact of *ABCB1* variants on long-term MACE may be modest or population-specific.

Yet, some studies have also examined the link among the three *ABCB1* variants and bleeding outcomes in patients with ACS using clopidogrel as an antiplatelet. A study established a significant link between the *ABCB1* rs1045642 (c.C3435T) variant and bleeding risk and outcomes (42, 47). Moreover, it has been established that the presence of the variant allele of the rs102032582 (c.2667T) polymorphism correlates with the risk of the development of overall bleeding outcomes without affecting the residual platelet reactivity (RPR) (48). The findings warrant further assessment of the UAE population to establish correlations with clopidogrel safety.

With regard to *CYP2C19* variants, CYP2C19*3 exhibited a low frequency in the current cohort (1.15%), which is consistent with previous reports from UAE citizens (20) and other admixed populations (22, 49). These findings confirm the evidence that CYP2C19*3 is a rare LOF allele among the Middle East populations. In contrast, CYP2C19*2 exhibited a much higher frequency among patients with ACS (25%), and the variant demonstrated a trend toward a higher risk of MACE that did not reach statistical significance (RR = 1.41; 95% CI: 0.95–2.10; *p*-value = 0.08). It is further reinforced by the well-established guidelines that *CYP2C19* is the primary target for the current genotyping recommendations for antiplatelets (28).

In the logistic regression analysis, it was observed that neither the three common *ABCB1* variants nor the clinical factors were significant predictors of incidence of MACE in patients with ACS. This suggests that a larger patient cohort must be studied to better predict the factors that modulate the risk of the development of MACE in patients with ACS.

Several factors may explain the negative findings. Clopidogrel response is multifactorial and the findings should be interpreted with caution as type II error cannot be excluded. First, the study participants were not only exclusively receiving clopidogrel, as many patients were concurrently treated with cardiovascular drugs and other drugs like PPIs (78%), which may have influenced the PK and pharmacodynamics (PD) responses, thus confounding the results. In addition, the UAE population is highly mixed and this genetic heterogeneity may have contributed to the variability in allele distribution and drug response. Stratification by ethnicity was not feasible due to the limited sample size, which may have obscured population-specific effects.

Other factors, including the relatively small sample size, may have limited the statistical power of the study. A larger sample size would have increased the reliability of the results, allowing for more thorough and precise conclusions.

In conclusion, this study investigated the relationship of *CYP2C19* and *ABCB1* variants with clopidogrel efficacy in patients with ACS from the UAE. Common *ABCB1* polymorphisms have limited effect on clopidogrel response and MACE risk. These findings highlight the multifactorial nature of clopidogrel response and suggest that other genetic and/or environmental factors may play a more important role. Future studies evaluating additional pharmacogenomic markers with better control of confounding factors are needed in the admixed UAE population.

## Data Availability

The data presented in this study are not publicly available due to participant privacy concerns and data sharing restrictions set by the institutional ethics board. De-identified data may be made available upon reasonable request to the corresponding author, subject to ethical approval.
